# Supplementary material for: Pharmacological Potential and Chemical Composition of Crocus sativus Leaf Extracts
Source: Molecules. 2021 Dec 21;27(1):10. doi: 10.3390/molecules27010010 (PMC8746171; doi:10.3390/molecules27010010)
Supplement: Supplementary file 1 [file molecules-27-00010-s001.zip › molecules-1484075-supplementary.pdf]

Supplementary Material

Pharmacological potential and chemical composition of *Crocus sativus* leaf extracts

Olha Mykhailenko [1,†], Vilma Petrikaitė [2,3,†], Michal Korinek [4,5,6,7], Fang-Rong Chang [4], Mohamed El-Shazly [8,9,*], Chia-Hung Yen [4], Ivan Bezruk [1], Bing-Hung Chen [5,10], Chung-Fan Hsieh [11], Dmytro Lytkin [12], Liudas Ivanauskas [13], Victoriya Georgiyants [1] and Tsong-Long Hwang [6,7,14,15,*]

Table of Contents

Table S1. Calibration curves of the reference standard compounds. 2

Table S2. Precision and stability of the quantified compounds. 3

Table S3. The specificity of the quantified compounds. 4

Materials and Methods 5

Table S1. Calibration curves of the reference standard compounds.

Compound		Calibration curve[a]	Correlation coefficient r^2 (n = 6)	Linear range (μm/mL)	RSD, %	LOD[b] (ng/mL)	LOQ[c] (ng/mL)
1	Chlorogenic acid	Y = 29930.2x-538.361	0.9999502	0.36-46	1.29	20	70
2	Caffeic acid	y = 57646.8x-3853.48	0.9999218	0.72-91.92	1.56	20	60
3	Mangiferin	y = 29263.5x+13863.9	0.9997952	0.28-145.00	1.32	310	940
4	Isoorientin	y = 26559.9x+2849.65	0.999996	0.73-92.85	1.41	8	24
5	Ferulic acid	y = 54955.4x-638.345	0.9999592	0.44-56.5	1.60	30	80
6	Rutin	f(x) = 16072.5x+1499.73	0.9998787	0.16-20.24	1.07	96	290
7	Tectoridin	y = 76104.4x+114152	0.9995802	0.51-260.00	0.55	130	400
8	Quercetin	y = 39349.5x+1454.47	0.9998503	0.16-20.08	0.67	31	90
9	*t*-Cinnamic acid	y = 78502.3x+0	1.000000	0.3-38.75	0.12	30	90
10	Genistein 7-D-glucoside	y = 21546.1x+1464.76	0.9998262	0.09-12.02	0.59	70	210
11	Apigenin	y = 50138.3x+5722.97	0.9998899	0.2-25.76	0.53	25	80
12	Kaempferol	y = 29888.8x+1814.27	0.9999240	0.14-18.32	0.90	37	110
13	Iristectorigenin B	y = 109562x+68062.7	0.9996806	0.23-120.00	0.85	50	150
14	Nigricin	y = 89415.4x+103288	0.9994037	0.35-181.00	0.30	40	130
15	Irigenin	y = 81832.6x+137668	0.9994881	0.54-277.00	0.64	50	160
16	Biochanin A	y = 66391.3x+6173.76	0.9998366	0.15-18.76	1.12	32	100

[a]concentration of compound (mg/mL); y, peak area; [b]LOD, limit of detection (S/N = 3); [c]LOQ, limit of quantification (S/N = 10)

Table S2. Precision and stability of the quantified compounds.

	Compound	Concentration (μg/mL)	Precision				Repeatability	
			Intra-Day (*n* = 3)		Inter-Day (*n* = 3)		Recovery (%)	RSD (%)
			RSD (%)	Accuracy (%)	RSD (%)	Accuracy (%)		
1	Chlorogenic acid	5.75	1.31	101.12	0.38	98.40	100.69	0.86
		23	0.42	99.08	0.73	99.43	99.58	1.05
		46	0.96	100.27	0.48	98.24	101.91	0.97
2	Caffeic acid	11.49	1.05	102.02	0.52	98.49	100.01	0.46
		45.96	1.08	98.78	0.67	99.73	99.39	0.99
		91.92	0.64	100.35	0.95	98.17	100.17	0.37
3	Mangiferin	9.06	0.33	100.46	0.29	100.41	100.29	0.25
		36.25	0.24	99.66	0.32	100.45	100.03	0.39
		145	0.22	100.32	1.10	98.45	99.58	0.99
4	Isoorientin	0.73	0.37	101.15	0.72	101.97	101.17	0.88
		11.6	0.91	100.78	0.58	10.62	99.98	0.55
		46.42	0.45	100.15	0.67	99.92	100.03	0.27
5	Ferulic acid	7.06	0.68	100.22	0.90	98.29	99.11	0.69
		28.25	0.93	98.20	0.29	99.31	99.60	0.57
		56.5	1.22	100.24	0.46	98.28	100.12	0.49
6	Rutin	2.53	1.26	100.35	0.62	100.15	100.18	0.55
		10.12	1.29	101.12	0.80	99.21	100.65	0.92
		20.24	0.76	99.56	1.14	100.94	99.78	0.31
7	Tectoridin	16.25	1.35	101.93	1.57	102.24	101.39	0.98
		65	1.13	101.92	0.72	101.03	100.98	0.95
		260	0.30	99.57	0.03	99.96	99.84	0.23
8	Quercetin	2.51	1.07	100.71	0.33	98.81	99.86	0.80
		10.4	0.81	99.30	0.44	102.23	99.15	0.61
		20.08	0.64	100.27	0.57	100.22	100.14	1.07
9	*t*-Cinnamic acid	4.77	0.68	100.41	0.62	100.09	99.41	0.29
		21.01	1.09	99.55	0.80	100.97	99.55	0.31
		38.795	1.10	98.99	0.47	101.53	99.99	0.56
10	Genistein 7-D-glucoside	1.5	0.48	101.20	0.81	98.32	100.60	0.84
		6.01	0.66	100.51	0.61	98.02	100.76	0.66
		12.02	0.87	99.48	0.49	100.02	99.74	0.37
11	Apigenin	4	0.74	100.75	0.57	98.76	100.56	0.79
		16	0.89	100.89	0.29	98.62	98.96	0.71
		32	0.88	100.70	0.70	98.03	99.80	1.02
12	Kaempferol	2.29	0.48	100.69	0.44	99.81	100.35	0.49
		9.16	0.77	100.97	1.01	99.54	100.49	0.68
		18.32	1.14	99.66	1.12	100.84	99.83	0.34
13	Iristectorigenin B	7.5	1.23	101.76	1.64	102.35	101.36	0.97
		30	1.01	102.88	1.23	101.76	101.54	1.01
		120	0.07	99.90	0.33	99.53	99.81	0.25
14	Nigricin	11.31	1.19	101.70	1.21	101.73	101.14	0.98
		45.25	0.37	99.47	1.19	101.70	100.39	0.96
		181	0.57	99.19	0.48	99.33	99.50	0.43
15	Irigenin	17.31	1.08	101.54	1.29	101.84	101.12	0.98
		69.25	0.80	101.14	1.16	101.65	100.93	0.84
		277	0.33	99.53	0.20	99.71	99.74	0.24
16	Biochanin A	2.35	0.61	100.72	0.45	98.79	99.36	0.51
		9.38	0.80	100.47	0.60	98.06	100.74	0.53
		18.76	0.67	99.49	0.77	100.00	99.75	0.66

Table S3. The specificity of the quantified compounds.

**Chlorogenic acid**[a]	**Caffeic acid**	**Mangiferin**	**Isoorientin**	**Ferulic acid**	**Rutin**
310 nm[b] and 11.66 min[c]	310 mn and 14.18 min	270 mn and 14.18 min	310 mn and 17.66 min	310 mn and 21.64 min	310 nm and 22.48 min
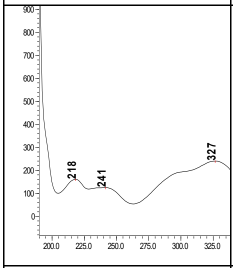	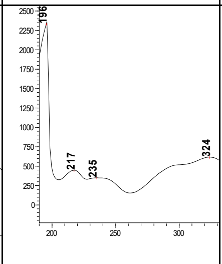	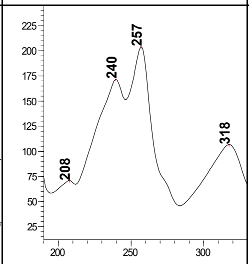	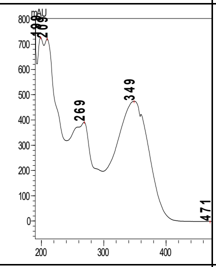	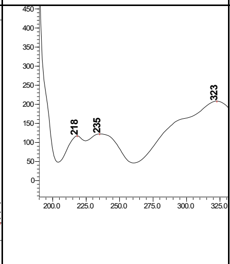	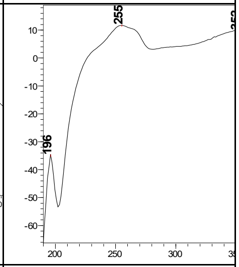
**Tectoridin**	**Quercetin**	**Irigenin**	**Iristectorigenin B**	**Nigricin**	***t*-Cinnamic acid**
269 nm and 29.89 min	310 nm and 43.71 min	269 nm and 50.03 min	269 nm and 49.15 min	269 nm and 49.50 min	270 nm and 45.22 min
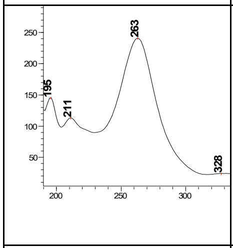	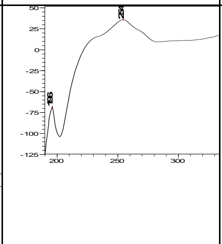	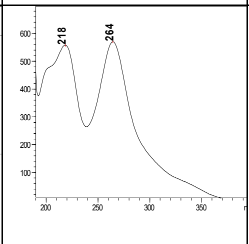	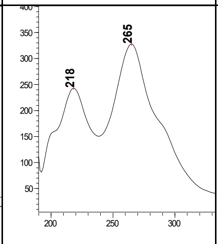	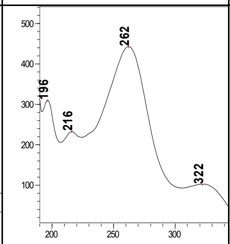	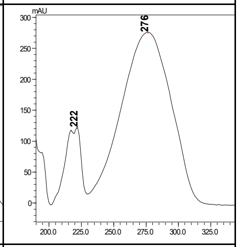
**Genistein 7-D-glucoside**	**Apigenin**	**Kaempferol**	**Biochanin A**		
260 nm and 46.07 min	340 nm and 47.90 min	370 nm and 48.99 min	260 nm and 60.66 min		
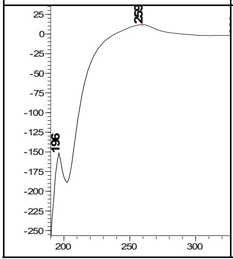	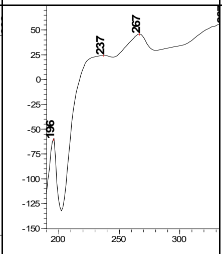	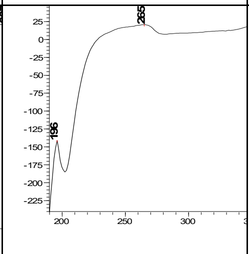	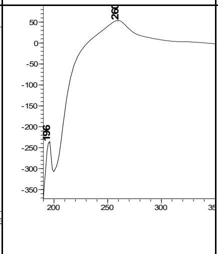		

Note: [a]Compound; [b]Detected at wavelength, λ, nm, c – Retention time, min

Materials and Methods

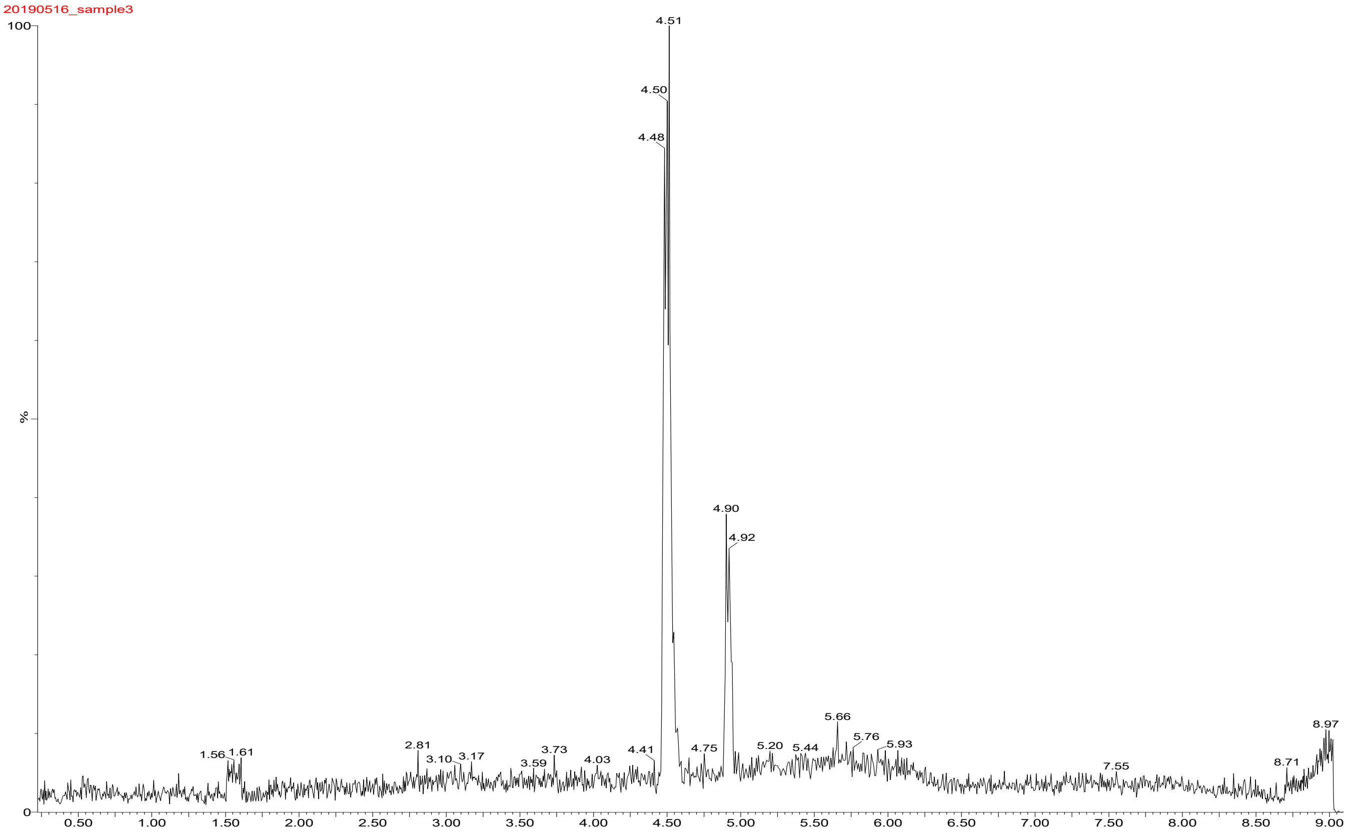

Figure S1. The UPLC-MS/MS-chromatogram of *C. sativus* leaves aqueous extract

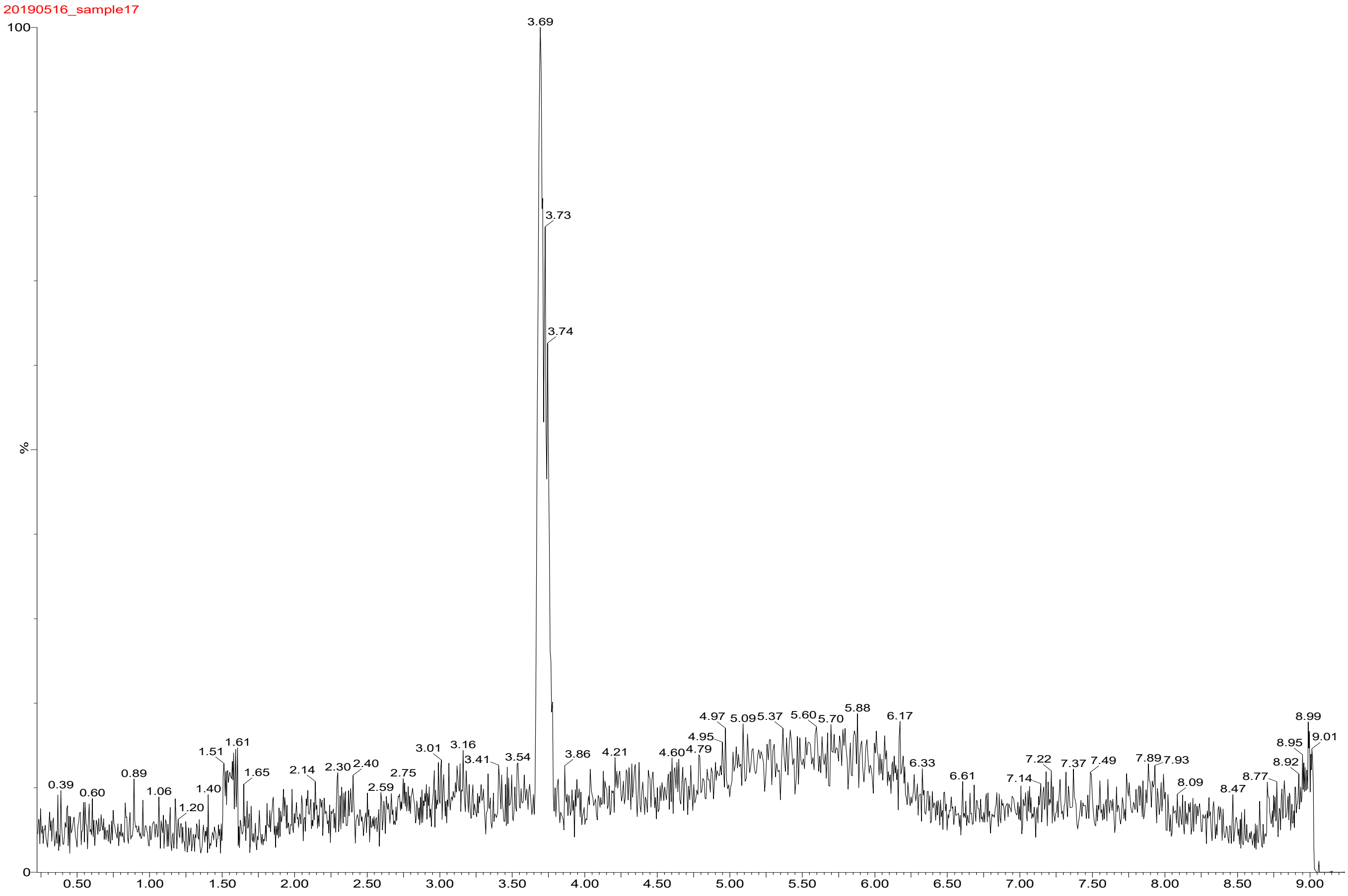

Figure S2. The UPLC-MS/MS-chromatogram of *C. sativus* leaves ethanolic extract

UPLC-MS screening

Following is a brief explanation for the identification of the compounds reported in Figure 1 and Table **2** of the main text and Figure 1S and 2S of Supplementary material.

Four hydroxycinnamic acid derivatives namely caffeic acid, chlorogenic acid, cinnamic acid and ferulic acid were identified by comparing their retention times and characteristic MS spectral data with those of authentic standards (**Table S2**). Accurate mass measurements and fragmentation pattern further confirmed their structural composition. The pseudomolecular ion of compound **E** with $[M-H]^-$ ions m/z 193 produced the major fragment ions at m/z 149 corresponding to the loss of carbon dioxide from the precursor ion. The other fragment of compound **E** was at m/z 178 due to initial loss of a methyl group from the precursor ion. Compound **E** was identified as a ferulic acid. The remaining two hydroxycinnamic acid derivatives; caffeic acid, cinnamic acid and monocaffeoyquinic acid were identified by their accurate mass measurements and MS/MS spectral data. Compound **A** with $[M-H]^-$ at m/z 353 was assigned to monocaffeoyquinic acids. The MS spectrum of this compound was characterized by the loss of one moiety of caffeic acid, due to the ester bond, which readily dissociated and was identified as 3-O-caffeylquinic acid or chlorogenic acid in comparison with the reference standards.

The tentative mass spectrum for compound **B** showed the deprotonated molecule [M-H]- ion at m/z 179 at 3.92 min. The major fragment ions were m/z 161 and m/z 135 corresponding to loss of water and carbon dioxide molecules respectively from the precursor ion. Compounds **B** with $[M-H]^-$ ions at m/z 179 was identified as caffeic acid (Jeong et al., 2020). The compound I with $[M-H]^-$ m/z 147 at 6.80 min was identified as *trans*-cinnamic acid. Based on the fragmentation pattern described by Schaldach and co-authors (Schaldach et al., 1980), the fragment ion observed at m/z 119 corresponded to $[M-H-CO]^-$. Similar pattern of fragmentation of hydroxycinnamic acid derivatives has been reported by several authors (Spínola et al., 2018; Hossain et al., 2010; Sun et al., 2007).

Mangiferin (compound **C**) was the only *C*-glycosidic xanthone derivative identified in *Crocus* leaves extracts. by this method. In the MS spectrum compound **C** produced the $[M – H]^–$ ion at m/z 421. The MS2 spectrum gave the predominant fragment ions at m/z 331 and 301 owing to the neutral loss of $C_3H_6O_3$ and $C_4H_8O_4$, respectively. The low intensity product ion at m/z 259 appeared in the MS2 spectrum due to the loss of the glucose residue from the $[M – H]^–$ ion. The MS3 spectrum of the precursor ion at m/z 301 gave the product ion at m/z 273, 271 and 257 resulting from the loss of CO, CH_2O and CO2, respectively. The RDA fragmentation reaction was also observed in the MS/MS spectrum and corresponding with literature data (Yu et al., 2013).

Flavonoids are the most abundant substances in *Crocus* leaves extracts. With the aid of reference standards and complemented by the accurate mass measurement data, eight flavonoids were identified. The five flavonoids were apigenin, isoorientin, kaempferol, quercetin and rutin. Furthermore, the fragmentation

pattern of these flavonoids was similar to those described previously where the most common fragment lost was a water molecule and a glucose moiety in the two glucosides.

For flavones, compound **L** [M-H]$^-$ peak at m/z 269 at 6.22 min, compound **H** [M-H]$^-$ at m/z 301, and compound M [M-H]$^-$ at m/z 285 were identified as apigenin (calculated for $C_{15}H_{10}O_5$, m/z 270), quercetin (calculated for $C_{15}H_{10}O_7$, m/z 302), kaempferol (calculated for $C_{15}H_{10}O_6$, m/z 286), respectively. Compound **F** showed a [M-H]- peak at m/z 609 at 4.82 min, and had the deprotonated [M-H]- molecule of the glycoside and the [A-H]- ion corresponding to the deprotonated aglycone. The latter ion is formed by losing the rutinose moiety from the corresponding glycoside. Compound **F** was identified as quercetin-O-rutinoside (rutin) (Jeong et al., 2020). Compound **D** with [M-H]$^-$ at m/z 447 was identified as isoorientin (luteolin 6-C-glucopyranoside). The presence of a fragment ion at m/z 429 [M-H-18]$^-$ is characteristic of isoorientin. They were further confirmed by comparing the retention time and the MS/MS spectra with the corresponding standards. Our studies are consistent with the authors' data (Hossain et al., 2010; Chen et al., 2016; Luo et al., 2020; Zhao et al., 2020) on the fragmentation of flavones.

Compounds **G**, **K**, **N**, **O**, **P** demonstrated maximum absorption peaks at 218-345 nm (shoulder peak) which are characteristic peaks of isoflavones. MS data were measured in the negative ion mode and the mass spectroscopic data of all compounds are listed in Table **2**. The detected compounds demonstrated regular MS fragmentation behavior, which was useful in providing information on their chemical structures. The MS spectra of flavonoid glycosides exhibited a loss of 162 Da, suggesting the presence of one hexose residue. This fragmentation pattern was characteristic of O-glycosides, such as compound **G** (tectoridin) and compound **K** (genistein-D-glucoside). The loss of a methyl radical ion (15 Da) was the predominant fragmentation pattern for most of the compounds, owing to the loss of a methoxy group. Compound **K** displayed [M-H]$^-$ ion at m/z 431 and was identified as a genistein-7-glucoside according to a previous report (March et al., 2004). A fragment ion was generated at m/z 269 after the loss of an hexose moiety from the parent ion. For example, compounds **N** (iristectorigenin B) and compound P (biochanin A) exhibited an ion peak at m/z 329 and m/z 283, respectively in the negative ion mode. The mass data showed a fragment ion at m/z 314 and m/z 268 indicating the loss of a methyl residue. Compound **O** (irigenin) with [M-H]$^-$ ion at m/z 359 lost three methyl groups showing fragments at m/z 344 and m/z 329. The isoflavonoid nigricin is identified in the positive ionization mode according to the retro-Diels-Adel reaction (RDA) (Shu et al., 2010). Since we were able to identify almost all substances in the negative ion mode, we did not carry out a separate determination for nigricin.

Details of procedures from Section 3 of main text are described below, including HPLC methodology and bioactivity assays.

3.9. HPLC-PDA and UPLC-MS conditions, HPLC post-column assay

Waters Alliance 2695 (Waters, Milford, USA) separation module system equipped with Waters 2487 UV/VIS and Waters 996 PDA diode-array detector (DAD) were used. HPLC grade water was obtained from a water purifying system (Millipore, Bedford, MA, USA). 2,2′-Azino-bis (3-ethylbenzothiazoline-6-sulfonic acid) diammonium salt (ABTS, purity ≥ 98%), potassium persulphate (purity ≥ 99 %) were purchased from Sigma (St. Louis, MO, USA), and Trolox (purity ≥ 98%) from Fluka Chemika (Buchs, Switzerland). The compounds separation was carried out utilizing an ACE C18 column (250 mm × 4.6 mm, 5.0 μm, Pennsylvania, USA) with a YMC guard cartridge ODS-A (3.0 μm, 10 mm × 4.0 mm). The binary solvent system of the mobile phase comprised solvent A (0.1% acetic acid in water) and solvent B (acetonitrile). A following linear gradient program was applied: 0–8 min, 5–15% B; 8–30 min, 15–20% B; 30–48 min, 20–40% B; 48–58 min, 40–50% B; 58–65 min, 50%; 65–66 min, 50–95% B. The injection volume of the sample solution was 20 μL. After PDA detection, the ABTS solution was mixed with the mobile phase carrying the analytes in the reaction coil (Raudonis et al., 2009; Raudonis et al., 2012). Empower Software Chromatographic Manager System (Waters Corporation, Milford, USA) was used to analyze the data. The ABTS post-column chromatograms were detected at the wavelength of 650 nm using Waters 2487 UV/VIS detector (Waters Corporation). The standard antioxidant Trolox (0.3995 μmol/g) was used for the preparation of the calibration curves (Marksa et al., 2016). Trolox equivalent antioxidant capacity (TEAC) was used to express antioxidant activity. The value was calculated as μmol Trolox equivalent (TE) for 1 g of dry mass (DM) of the plant material using the following formula: TEAC = c × V/m (μmol/g) where 'c' is the Trolox concentration in μM established from the calibration curve, 'V' is the plant material extract volume in L, and 'm' is the weight (precise) in g.

Separation of the samples' components was carried out with the ACQUITY H-class UPLC system (Waters, Milford, MA, USA) equipped with ACQUITY UPLC BEH C18 (50 × 2.1 mm, particle size 1.7 μm) (Merck Millipore, Darmstadt, Germany). Gradient elution was performed with 0.1% formic acid water solution (solvent A) and acetonitrile (solvent B), the flow rate at 0.5 mL/min. The following proportions of the solvent system were applied using a linear gradient profile B: Initial 0-3 min, 5%, 3-10 min, 30%, 50%, 10-18 min, 95%, 18-34 min, 5%. Xevo TQD triple quadrupole mass spectrometer detector (Waters) was used to obtain MS/MS data. Positive electrospray ionization was applied with the following settings: Capillary voltage was 1.5 kV, source temperature was 150 °C, desolvation temperature was 350 °C, with a desolvation gas flow 650 L/h, cone gas flow was 25 L/h. Collision energy and cone voltage were optimized for each compound separately. Collision energy varied in the range from 6eV to 20 eV and cone voltage was selected from 8 V to 38 V.

3.12. In-vitro assessment of NRF2 activity

The cell line HaCaT/ARE was developed from a HaCaT stable cell line carrying a fragment derived from pGL4.37[luc2P/ARE/Hygro] plasmid that contains four copies of an antioxidant response element (ARE) that drives the transcription of the luciferase reporter gene luc2P. The reporter cells were cultured in Dulbecco's Modified Eagle's Medium (DMEM) (Gibco BRL, Grand Island, NY, USA) supplemented with penicillin (100 U/mL), streptomycin (100 μg/mL), 10% heat-inactivated fetal bovine serum (HyClone, Logan, UT, USA), and 100 μg/mL hygromycin. The cells were maintained in a humidified incubator with 5% CO_2. Reporter cells were seeded (1×10^4 cells/well) in 96-wells plate, then were

treated with the indicated concentration of the sample for 18 h (single measurement). Resazurin (Cayman Chemical, Ann Arbor, MI, USA) with a final concentration of 0.1 mg/mL was added and the cells were incubated for an additional 4 h at 37 °C. Fluorescence of the reduced resazurin in the supernatant of the cell (ex/em: 530 nm/590 nm) was detected using a Synergy HT Multi-Mode Reader (BioTek, Winooski, VT, USA) to determine cell viability. The cells were then harvested and luciferase activity measurements were achieved according to the manufacturer's protocol (Promega Corporation, Madison, WI, USA). The luciferase activity was normalized to cell viability and the relative luciferase activity was calculated and compared with the control (DMSO).

3.13. Lipid droplet assay

Lipid droplet accumulation was established by treating Huh7 cells with BSA-conjugated oleic acid. Cells were seeded in µClear® 96-wells plates (Greiner Bio-ONE, Frickenhausen, Germany) and treated with oleic acid and the tested drugs or DMSO for 18 h. Paraformaldehyde was used to fix the cells, which were stained with 2 µg/mL Hoechst 33342 and 1 µg/mL BODIPY® 493/503 HCS instrument was used to take and analyze images of the nuclei and lipid droplets (ImageXpress Micro System, Molecular Devices, Sunnyvale, CA, USA). The diameter settings were 8–25 µm for the nuclei and 0.5–2 µm for the lipid droplets.

3.14. In vitro assessment of the anti-allergic activity

3.14.1. Chemicals and reagents

Dulbecco's modified Eagle's medium-high glucose powder (DMEM), [3-(4,5-dimethylthiazol-2-yl)-2,5-diphenyltetrazolium bromide] (MTT), *p*-nitrophenyl-*N*-acetyl-D-glucosaminide (p-NAG), penicillin and streptomycin, dexamethasone, calcium ionophore A23187, mouse anti-DNP IgE antibody, and dimethyl sulfoxide (DMSO) were purchased from Sigma-Aldrich (St. Louis, MO, USA). Moreover, fetal bovine serum (FBS) was obtained from Hyclone (Logan, UT, USA). Dinitrophenyl-conjugated bovine serum albumin (DNP-BSA) was purchased from Merck (Kenilworth, NJ, USA). All other chemicals and reagents were purchased at the highest possible purity and quality.

3.14.2. Cell Culture

The mucosal mast cell-derived rat basophilic leukemia (RBL-2H3) cell line was purchased from the American Type Culture Collection. Cells were grown in DMEM medium supplemented with 10% FBS and 100 U/mL penicillin plus 100 µg/mL streptomycin. Cells were cultured in 10 cm cell culture dishes (Cellstar) at 37 °C in a humidified chamber with 5% CO_2 in the air.

3.14.3. Cell viability assay

The degree of cell viability of each sample was calculated as the percentage of the control value (untreated cells). All experiments were repeated three times. The maximally tolerated dose of DMSO was 0.5%. It served as control not affecting RBL-2H3 cell growth. Triton X-100 (0.5% solution) was used as the positive control causing the death of all cells in a well.

3.14.4. Degranulation β-hexosaminidase assay induced by A23187 and antigen

RBL-2H3 cells were dispensed into the 96-wells plate at a density of 2×10^4 cells/well (A23187-induced assay) or 48-wells plate at a density of 3×10^4 cells/well (antigen-induced assay). Cells were incubated at

37 °C in 5% CO_2 for at least 5 h to allow the cells to completely adhere to the bottom of the wells. Cells were washed with PBS and various concentrations of the samples or medium (untreated control) were added to each well (100 µL), followed by 20 h of incubation at 37 °C in 5% CO_2. Dexamethasone (10 nM) was used as a positive control. The cells for antigen-induced assay were sensitized with anti-DNP IgE (0.1 µg/mL) for 2 h. Afterwards, the cells were washed by pre-warmed Tyrode's buffer (135 mM NaCl, 5 mM KCl, 1.8 mM $CaCl_2$, 1.0 mM $MgCl_2$, 5.6 mM glucose, 20 mM HEPES, and 1 mg/mL BSA at pH 7.4) and were stimulated by calcium ionophore A23187 (1 µM) or cross-linking antigen DNP-BSA (100 ng/mL) diluted in Tyrode's buffer. The cells were incubated at 37 °C in 5% CO_2 for 1 h. Unstimulated cells were either lysed with 0.5% Triton X-100 solution for the total amount of *β*-hexosaminidase release or left untreated for the spontaneous release of *β*-hexosaminidase. Stimulated but untreated cells served as the control. Then aliquots of the supernatants (50 µL) collected from the control and experimental wells were incubated with an equal volume (50 µL) of 1 µM of *p*-NAG (*p*-nitrophenyl-*N*-acetyl-*β*-D-glucosaminide) prepared in 0.1 M citrate buffer (pH 4.5) serving as the substrate for the released *β*-hexosaminidase. After 1 h of incubation at 37 °C, the reaction was quenched by the addition of 100 µL of stop buffer (0.1 M $Na_2/NaHCO_3$, pH 10.0). Absorbance was measured at 405 nm on a microplate reader. The inhibition percentage of *β*-hexosaminidase release from RBL-2H3 cells was calculated as the percentage of the control value (untreated stimulated cells) using the following equation:

$$\text{Inhibition}\,(\%) = \left[1 - \frac{(\text{ODsample} - \text{ODspontaneous})}{(\text{ODcontrol} - \text{ODspontaneous})}\right] \times 100$$

3.15. In vitro assessment of the anti-inflammatory activity

3.15.1. Preparation of human neutrophils

Blood was taken from healthy human donors (20-35 years old) by venipuncture using a protocol approved by the institutional review board at Chang Gung Memorial Hospital. Neutrophils were isolated using a standard method as previously described (Boyum, 1968).

3.15.2. Measurement of superoxide generation

Neutrophils (6 × 10^5/mL) supplemented with 0.5 mg/mL ferricytochrome *c* and 1 mM Ca^{2+} were equilibrated at 37 °C for 2 min and then incubated with the tested compounds or DMSO (control) for 5 min. Genistein served as a positive control. Cells were activated with formyl-methionyl-leucyl-phenylalanine (fMLF, 100 nM)/cytochalasin B (CB, 1 µg/mL) for 10 min. The absorbance was continuously monitored at 550 nm in a double-beam, six-cell positioned spectrophotometer Hitachi U-3010 with constant stirring (Hitachi Inc., Tokyo, Japan). Calculations were based on the differences in absorbance with and without superoxide dismutase (SOD, 100 U/mL) divided by the extinction coefficient for the reduction of ferricytochrome *c* (ε = 21.1/mM/10 mm).

3.15.3. Measurement of elastase release

Neutrophils were equilibrated with MeO-Suc-Ala-Ala-Pro-Val-p-nitroanilide (100 µM), an elastase substrate, at 37 °C for 2 min and then incubated with the drugs for 5 min. Genistein served as a positive control. Cells were activated by 100 nM fMLF and 0.5 µg/mL CB, and changes in the absorbance at 405 nm were continuously monitored to monitor the elastase release. The results were expressed as the percent of the initial rate of elastase release in the fMLF/CB-activated drug-free control system.

3.16. In vitro cytotoxic activity

3.16.1. Cell culture

Human melanoma cancer cell line IGR39 and human triple-negative breast cancer cell line MDA-MB-231 were obtained from the American Type Culture Collection (ATCC, Manassas, VA, USA). Cells were grown in DMEM Glutamax medium (Gibco, Carlsbad, CA, USA) containing 10% fetal bovine serum and 1% antibiotic mixture (10,000 U/mL penicillin and 10 mg/mL streptomycin; Gibco). All cells were incubated at 37 °C in a humidified atmosphere containing 5% CO_2.

3.16.2. Cell viability assay

IGR39 and MDA-MB-231 cells were seeded (3,000 and 5,000 cells/well, respectively) in 96-well plate and incubated overnight at 37 °C in a humidified atmosphere containing 5% CO_2. Cells were affected by various concentrations of the tested extracts (from 1 mg/mL to 31.25 µg/mL). As a background control, the only medium without cells was used and the medium with 0.4% ethanol served as the negative control. After 72 hours of incubation with the extracts, 20 µL of MTT (5 mg/ml) was added into each well and incubated for 4 hours under the same conditions. The supernatant was removed and 100 µL DMSO was added. The absorbance was measured at 570 nm and 630 nm, and EC_{50} (half-maximal effective concentration of a drug at which 50% of its maximum response is observed) values were calculated.

2.13. Protective effect of the extracts against influenza virus and enterovirus

The anti-viral assay was performed by cytopathic effects of the extracts on the cells infected by influenza H1N1 [32], and enterovirus D68 [33]. Briefly, the 96-well tissue culture plates were seeded with MDCK cells (2×10^4 per well) or RD cells (2×10^4 cells /well) in E10 medium (DMEM containing 10% FBS, 100 U/mL penicillin (Gibco, USA), 100 µg/mL streptomycin (Gibco, USA), 2 mM L-glutamine (L-glutamine) (Gibco, Brazil), 0.1 mM nonessential amino acid mixture (NEAA, Gibco, USA) and were incubated under 5% CO_2 for 16–24 h at 37 °C. The culture medium was withdrawn, and the wells were washed once with Dulbecco's phosphate-buffered saline (DPBS). The cells were infected with influenza virus (A/WSN/33) or enteroviruses at a nine-fold median tissue culture infective dose, with or without the addition of the samples. The treated cells were further incubated for 72 h at 37 °C. After 72 h, the medium was removed, and the cells were fixed with 4% paraformaldehyde for 1 h at room temperature. Then, 0.1% crystal violet was used to stain the cells for 20 min at room temperature. The cells density was measured using a VICTOR3™ multilabel plate reader (PerkinElmer).

2.14. Coronavirus 229E assay

The protective effect of the samples against human coronavirus 229E (HCoV-229) was determined according to a previously described method [34]. Huh7 cells (human liver carcinoma cell line) were infected with 9 times $TCID_{50}$ (Median Tissue Culture Infectious Dose) of each coronavirus 229E in the presence or absence of the compounds or vehicle. Incubation was done at 33 °C for 6 days, the surviving cells were then stained with MTT (3-[4.5-dimethylthiazol-2-yl]-2,5-diphenyl tetrazolium bromide). The percentage of surviving cells was then calculated.

2.15. Neuraminidase activity assay

A baculovirus displayed neuraminidase NA9 on the surface (NA9-Bac) was used to evaluate the neuraminidase activity. An appropriate virus load of NA9-Bac was added into a 96-well plate and was incubated with the extracts or compounds for 20 minutes at 37 °C. Each well was supplemented with 25 μL of the diluted fluorescent MUNANA substrate. After incubation for 30 minutes at ambient temperature, 150 μL of the stop solution was added. The fluorescence intensity was immediately detected using Synergy HT Multi-Mode Microplate Reader (BioTek). Zanamivir, a known neuraminidase inhibitor, was used as the positive control in this assay.
